# Acute perchloroethylene intoxication in an elderly woman: a case report

**DOI:** 10.1186/s13256-022-03631-0

**Published:** 2022-10-26

**Authors:** Alessandro Tarditi, Leda Montalbano, Stefano Spina, Francesco Marrazzo, Giampaolo Casella, Paolo Schenardi, Tommaso Conti, Ilaria Angeli, Mauro Minoli, Roberto Fumagalli, Thomas Langer

**Affiliations:** 1 School of Medicine and Surgery, University of Milano-Bicocca, Milan, Italy; 2 Department of Anesthesia and Critical Care, ASST Grande Ospedale Metropolitano Niguarda, Piazza Ospedale Maggiore 3, 20162 Milan, Italy; 3 Postgraduate School of Clinical Pharmacology and Toxicology, Università Degli Studi di Milano, Milan, Italy; 4 Poison Control Center, ASST Grande Ospedale Metropolitano Niguarda, Milan, Italy; 5 Dipartimento di Scienze Biomediche Chirurgiche ed Odontoiatriche, Laboratorio di Tossicologia Forense, Università Degli Studi di Milano, Via Luigi Mangiagalli, 37, 20133 Milan, Italy

**Keywords:** Acute intoxication, Poison control center, Coma, Critical care

## Abstract

**Background:**

Perchloroethylene is a colorless, strong-smelling substance commonly used for dry cleaning. Liver and kidney toxicities and carcinogenicity are well-known occupational hazards caused by chronic perchloroethylene exposure. Acute intoxication by ingestion of nondiluted perchloroethylene is rare in the adult population owing to its strong smell and taste. Very few data are available to physicians managing patients in this situation.

**Case presentation:**

An 89-year-old Caucasian woman accidentally drank perchloroethylene while visiting her laundry, leading to a coma within a few minutes. The poison control center provided little information about perchloroethylene toxicity after ingestion, including an estimated long biological half-life (144 hour) and detrimental effects to liver and kidneys. A long intensive care unit stay was thus expected, potentially leading to several complications. After intubation, transitory hypoxemia appeared and rapidly resolved, while mild hemodynamic instability was managed with fluid resuscitation and anti-arrhythmic drugs. Twelve hours after perchloroethylene ingestion, the patient suddenly woke up and self-extubated. Less than 24 hours after ingestion, she was discharged from the intensive care unit, and 4 days later she was discharged home.

**Conclusion:**

The patient drank perchloroethylene from a bottle, which prevented her from smelling it, and owing to its taste, only a small sip was likely drunk. However, a much larger intake was presumed, given her rapid and profound central nervous system depression. This case was challenging owing to the paucity of information available regarding acute perchloroethylene ingestion and the duration and magnitude of its effect. The present report will hopefully be of support for clinicians managing patients with this rare acute intoxication.

## Background

Perchloroethylene (PCE), also known as tetrachloroethylene, is a colorless substance with a strong odor—described as sharp, sweet, ether-like, similar to chloroform—with a density slightly higher than water, which is widely used for dry cleaning [1]. It was also used as an antiparasitic medication [2], although this is not the case anymore in Western countries. Its toxicity is well known, but PCE intoxication is mostly described and studied in three aspects: chronic inhalation exposure, which is a well-known occupational hazard in the dry-cleaning field [1]; acute inhalation, including substance abuse among young people owing to some degree of excitatory effect on the central nervous system (CNS), leading to euphoria [3, 4]; and ingestion of contaminated water from dry cleaning and metal degreasing industries leaching into underground water sources [5]. The latter is gaining attention as an environmental hazard owing to both the carcinogenicity [6] (the International Agency for Research on Cancer [IARC] has classified PCE in group 2A with a positive association with bladder cancer in humans, while further data are needed on possible associations with cancers of the esophagus, cervix, kidney, and non-Hodgkin lymphomas [7]) and neuro–behavioral effects [8] of chronic exposure.

The National Institute for Occupational Safety and Health of the American Center for Disease Control and the Agency for Toxic Substances and Disease Registry (ATSDR) of the US Department of Health and Human Services both produced specific guidelines concerning the toxicity and safety levels of PCE, focusing on chronic exposure at the working site and acceptable levels over an 8-hour shift [1, 5]. The ATSDR described a minimal risk level of 0.006 ppm for inhalation exposure and 0.008 mg/kg/day for chronic ingestion. Concentrations inducing narcosis and lethal concentrations are based on rat models: anesthetic effects appeared after acute exposure to 1750–2000 ppm and neurological effects after oral exposure to 5 mg/kg/day [5]. Available data from mice on acute ingestion of undiluted PCE indicate a lethal dose (LD)_50_ of 8.196 mg/kg [5]. Finally, observing the ATSDR tables for the no observed adverse effect level (NOAEL) and lowest observed adverse effect level (LOAEL) for the oral route, data on single dose administration on humans come from just two studies from 1964 (Haerer and Udelman) and 1929 (Kendrick) [5].

Health hazards of PCE are mostly known and described for the damage produced by its metabolites to the liver [9, 10] and kidneys [10] in chronically exposed workers, as well as studies in rats and mice exposed to PCE via both inhalation and oral routes [5]. PCE is converted by the liver into thrichloroacetic acid and, to a lesser extent, dichloroacetic acid, the latter being considered the primary hepatotoxic metabolite [5]. Reversible kidney damage was shown in humans exposed to acute inhalation of PCE vapor [5]. In terms of cardiovascular effects, although a similar compound (trichloroethylene) has been associated with structural heart changes and cardiomyopathy [11], only a single case report associated chronic inhalation PCE exposure with nonmalignant arrhythmias [12], with animal studies on dogs unable to show a similar effect [5]. The extensive risks associated with PCE, which is still used by approximately 60% of dry cleaners in the USA, are pushing towards safer alternatives [13].

Acute intoxication via ingestion appears to be a rare occurrence, especially among adults: although colorless, PCE is readily distinguishable from water owing to its smell and taste. Case reports of PCE ingestion, therefore, mostly include pediatric patients. A specific search for case reports and other documents related to acute intoxication by ingestion in adults led to very few results, none of which could help in the rapid decision-making and ethical implications that the intensive care team faced in the case presented here. Moreover, our poison control center had very little data at the time on the half-life of PCE in blood and the expected duration of its effects. The most relevant information was the urinary biological half-life of 144 hour, estimated from the excretion of PCE metabolites into the urine of patients occupationally exposed by inhalation [14].

We describe here a case of acute intoxication due to the ingestion of PCE in an 89-year-old woman with a surprisingly fast and complete recovery.

## Case presentation

An 89-year-old Caucasian woman, accidentally drank a sip of PCE while visiting her laundry, which had been carelessly poured into a bottle of water. Her medical record included a surgical aortic valve replacement, triple coronary artery bypass graft surgery, and arterial hypertension. The family described her as being completely autonomous at home, as proved by the fact that she worked in her own dry cleaning shop until the age of 88.

The exact amount of ingested PCE is still uncertain owing to the high variability of product concentration and the proprietary formulas used by the different brands. However, the patient later described having just a sip before realizing, based on the taste, that it was not water. Around 10 minutes after ingestion she became drowsy and the emergency medical service was activated by her relatives.

**Fig. 1:**
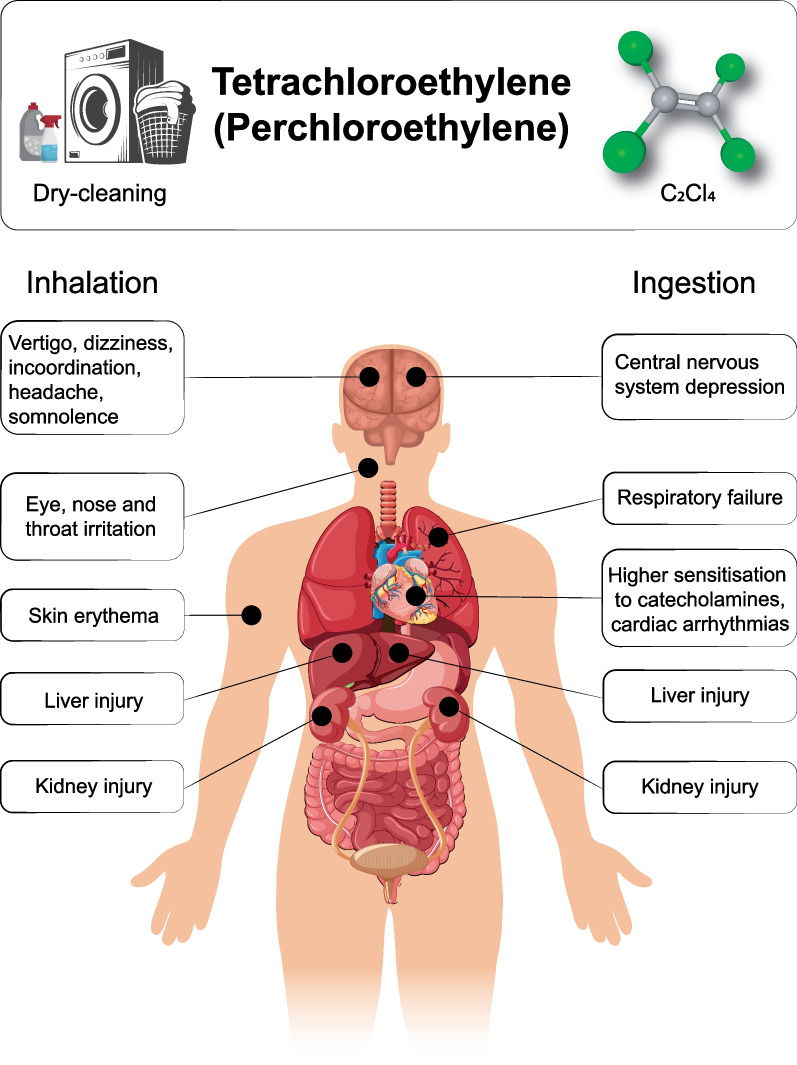
Overview of PCE characteristics and toxicity

When she arrived at the emergency room, approximately 50 minutes after the ingestion, her vitals were as follows: Glasgow Coma Scale (GCS) of 3, SpO_2_ 96%, respiratory rate of 34 breaths/minute with a snoring breathing, heart rate of 51 beats/minute, and blood pressure of 115/55 mmHg. The first blood-gas analysis did not show relevant abnormalities: pH of 7.38 and lactate 2.4 mmol/L. The poison control center was immediately contacted and promptly provided the available information regarding PCE toxicity: it is not caustic; it is a fat-soluble product, which tends to accumulate in the central nervous system (CNS); its half-life is extremely variable and uncertain in humans, with an estimated biological half-life of 144 hours; and its immediate life-threatening aspects are CNS and respiratory drive depression, and risk of cardiac arrhythmias, possibly owing to myocardial sensitization to endogenous and exogenous catecholamines. No antidote is available for PCE. Characteristics of PCE intoxication are summarized in Fig. 1.

The sudden and deep CNS depression led the whole team to presume that a large quantity of PCE had been ingested and that its effects on the CNS could presumably last for several days. This scenario would have led to prolonged mechanical ventilation, a long intensive care unit (ICU) stay, and a high risk of ICU-related complications, especially considering the patient’s advanced age.

After a team evaluation, the patient was intubated and admitted to the ICU. Intubation was devoid of any complication; however, the patient developed severe hypoxemia, with the need to increase FiO_2_ up to 1. After 4 hours of mechanical ventilation, the arterial partial pressure of oxygen was 70 mmHg with an FiO_2_ of 0.6 [ratio between partial pressure of oxygen and FiO_2_ (PaO_2_:FiO_2_) of 117]. Oxygenation improved in the following 3–4 hours, reaching an arterial partial pressure of oxygen of 155 mmHg at FiO_2_ of 0.6 (PaO_2_:FiO_2_ of 258).

The patient showed some hemodynamic instability and required fluid resuscitation with 3000 ml of crystalloids infused over the first 4 hours. The concentration of lactate was rapidly normalized and the patient had a normal urinary output. Five hours after the accidental ingestion, two episodes of supraventricular tachycardia occurred, promptly terminated with adenosine.

Initially, the patient remained comatose, with a GCS of 3. During the observation in the ICU, her neurological condition started to improve and the patient suddenly woke up and self-extubated herself 12 hours after the PCE ingestion (3 a.m.). Her respiratory function had recovered, she could cough properly and was completely awake.

At 8 a.m., her vital signs were as follows: SpO_2_ 98% with 2 L/minute nasal oxygen, respiratory rate of 17 breaths/minute, heart rate of 70 beats/minute despite a brief and self-limiting episode of atrial fibrillation, blood pressure of 145/50 mmHg, and urinary output 1400 ml from hospital arrival.

Less than 24 hours after ingestion she was discharged from the ICU, and 4 days later she was discharged from the hospital. No signs of liver or renal failure appeared, and the only complication during her hospital stay was a single episode of blood in the sputum. A blood and urine sample taken immediately after the discharge from the ICU was sent to the forensic toxicology department of the University of Milano for a quantitative measurement of PCE. The results were as follows: blood concentration 9.45 ng/ml and urine concentration 25.5 ng/mg.

## Discussion and conclusion

Acute intoxication by ingestion with PCE in adults appears to be a very rare event. Usually, the strong smell of PCE makes it very hard for adults to mistake it for water. Our patient, however, drank straight from the bottle and this, probably, prevented her from smelling it. Luckily, she immediately tasted something different and therefore probably drank only a small sip. However, this information was not available at the time of hospital admission, making it even harder for the treating physicians to estimate the expected duration and magnitude of PCE effects on the CNS and kidney and liver function of the patient.

Indeed, the profound CNS depression developing just a few minutes after drinking the product led the whole team to hypothesize a much larger intake. An additional challenge was the paucity of available information regarding the *in vivo* pharmacokinetics of PCE after ingestion of a single bolus. Although some pharmacokinetic models have been developed in the past decades [15], they lack the power to rapidly answer the main question the treating physicians had at the time: how long will the patient remain comatose?

Moreover, we had no information regarding the concentration of the ingested product (each dry cleaner usually has its own patented production process), and no immediate possibility to measure blood or urinary concentration. Indeed, after obtaining the ingested product from the dry cleaner, the laboratory needed a long processing time to perform a quantitative measurement. The only relevant information that the emergency team had on PCE was the estimated half-life of 144 hours; cardiac sensitization to amines, and thus high risk for cardiac arrhythmias; and the relevant hepatic and renal toxicity of the metabolites. The lack of an antidote and specific therapies to prevent such a detrimental failure of two vital organs led the team to expect the worst outcome. Moreover, the long half-life surely could imply several days of coma and mechanical ventilation.

However, reality proved all the expectations to be wrong, and the patient had a swift recovery of CNS function and never developed renal or liver injury. This rapid clinical course more accurately resembles the half-life estimated in another case report by Köppel *et al.* [16], where the PCE blood concentration profile was described by an open two-compartment model that showed a half-life of 160 minutes and 33 hours for the central and peripheral compartments, respectively. However, this information was not available at the onset of intoxication.

The patient was discharged from ICU on day 1 and from the hospital on day 5, in perfect physical and mental conditions. Cardiac sensitization showed itself in the hospital with two episodes of supraventricular tachycardia and a short episode of atrial fibrillation.

Chronic workplace exposure to the substance might also have enhanced her metabolism of PCE so that the half-life might be reduced for this reason. However, given the lack of liver and renal failure, it is conceivable to hypothesize that a small quantity of PCE was ingested.

The final, and probably most important, challenge that our team faced was ethical: based on the initial presentation, the advanced age, and the expected scenario, the hypothesis of withholding intensive care treatment was initially discussed. However, as any prognostic evaluation was necessarily based on a very weak scientific foundation, the team finally decided to proceed with a time-limited trial of intensive care treatment, with the idea of daily re-evaluating of the situation. This proved to be a good decision and we hope that this case report could help other emergency teams, should they face this rare intoxication.

Finally, our quantification of PCE in blood and urine is of little clinical application at the moment, as we were only able to acquire samples from a single time point. The very low levels compared with those described in ATSDR tables, however, further hint towards the ingestion of a very small amount [5]. Nevertheless, we decided to include them in this report as a possible reference for the future. In hindsight, measurement of PCE metabolites could have provided additional information; however, the satisfactory outcome of the patient at day 1 (the time of blood sampling) and our focus on the emergency and intensive care aspects of this case did not prompt such measurements.

## Data Availability

Not applicable.

## Appendix

PCE quantification was performed by extracting 0.5 ml of blood and 0.5 ml of urine in 10 ml vials, to which 0.5 ml of o-xilene was added. The vials were heated to 40 °C for 15 minutes, then 300 µL of air (head space) was collected with an air-tight syringe and injected in GC (Agilent 6890 coupled with a mass spectrometer Agilent 5975). The following ions were monitored: 166–129–94 m/z for PCE and 91–106 m/z for o-xilene. A sample of PCE, provided by the son of the patient from the laundry and corresponding to the same product the patient drank, was diluted 500 times and used for six calibration points by adding 0, 5, 10, 25, 50, or 100 µL of solution.

## Abbreviations

CNS: Central nervous system
GCS: Glasgow Coma Scale
ICU: Intensive care unit
PCE: Perchlorethylene
PaO_2_:FiO_2_: Ratio between the arterial partial pressure of oxygen and the inspired fraction of oxygen

## References

[1] “Occupational Health Guidelines for Tetrachloroethylene”—National Institute for Occupational Safety and Health, sept 1978.

[2] Bunnag D, Harinasuta T. Chemotherapy of intestinal parasites in south-east asia. Department of Clinical Tropical Medicine and Hospital for Tropical Diseases, Faculty of Tropical Medicine, Mahidol University, Bangkok, Thailand, p425, vol 12, no.3, Sept 1981

[3] Amadasi A, Mastroluca L, Marasciuolo L, Caligara M, Sironi L, Gentile G, *et al*. Death due to acute tetrachloroethylene intoxication in a chronic abuser. Int J Legal Med. 2015;129(3):487–93. 10.1007/s00414-015-1143-0. 25605280

[4] Isenschmid DS, Cassin BJ, Hepler BR, Kanluen S. Tetrachloroethylene intoxication in an autoerotic fatality. J Forensic Sci. 1998;43(1):231–4. 9456554

[5] Toxicological profile for tetrachloroethylene, Agency for Toxic Substances and Disease Registry (ATSDR) of the U.S. Department of Health and Human Services, June 2019, pages 10–11, 56–57, 119, 124. 37184172

[6] Paulu C, Aschengrau A, Ozonoff D. Tetrachloroethylene- contaminated drinking water in Massachusetts and the risk of colon- rectum, lung, and other cancers. Environ Health Perspect. 1999;107(4):265–71. 10.1289/ehp.99107265. PMC1566514 10090704

[7] IARC Monographs on the Evaluation of Carcinogenic Risks to Humans, volume 106, Trichloroethylene, Tetrachloroethylene, and some other chlorinated agents, 2014 PMC4781308 26214861

[8] Perrin MC, Opler MG, Harlap S, Harkavy-Friedman J, Kleinhaus K, Nahon D, *et al*. Tetrachloroethylene exposure and risk of schizophrenia: offspring of dry cleaners in a population birth cohort, preliminary findings. Schizophr Res. 2007;90(1–3):251–4. 10.1016/j.schres.2006.09.024. PMC2739584 17113267

[9] Shen C, Zhao CY, Liu F, Wang YD, Wang W. Acute liver failure associated with occupational exposure to tetrachloroethylene. J Korean Med Sci. 2011;26(1):138–42. 10.3346/jkms.2011.26.1.138. PMC3012839 21218043

[10] Lash LH, Parker JC. Hepatic and renal toxicities associated with perchloroethylene. Pharmacol Rev. 2001;53(2):177–208. 11356983

[11] Delepoulle F, Chauvière A, Brevière GM, Martinot A, Francart C, Diependaele JF, *et al*. Congestive cardiomyopathy after chronic inhalation of trichloroethylene. Arch Fr Pediatr. 1989;46(8):599–600. 2604515

[12] Abedin Z, Cook RC Jr, Milberg RM. Cardiac toxicity of perchloroethylene (a dry cleaning agent). South Med J. 1980;73(8):1081–3. 10.1097/00007611-198008000-00039. 7403923

[13] Ceballos D, Fellows K, Evans AE, Janulewicz PA, Lee EG, Whittaker SG. Perchloroethylene and dry cleaning: it’s time to move the industry to safer alternatives. Front Public Health. 2021;5(9): 638082. 10.3389/fpubh.2021.638082. PMC7973082 33748070

[14] Ikeda M, Imamura T. Biological half-life of trichloroethylene and tetrachloroethylene in human subjects. Int Arch Arbeitsmed. 1973;31(3):209–24. 10.1007/BF00539241. 4593976

[15] Chiu WA, Ginsberg GL. Development and evaluation of a harmonized physiologically based pharmacokinetic (PBPK) model for perchloroethylene toxicokinetics in mice, rats, and humans. Toxicol Appl Pharmacol. 2011;253(3):203–34. 10.1016/j.taap.2011.03.020. 21466818

[16] Köppel C, Arndt I, Arendt U, Koeppe P. Acute tetrachloroethylene poisoning–blood elimination kinetics during hyperventilation therapy. J Toxicol Clin Toxicol. 1985;23(2–3):103–15. 10.3109/15563658508990621. 4057308

